# The Indris Have Got Rhythm! Timing and Pitch Variation of a Primate Song Examined between Sexes and Age Classes

**DOI:** 10.3389/fnins.2016.00249

**Published:** 2016-06-14

**Authors:** Marco Gamba, Valeria Torti, Vittoria Estienne, Rose M. Randrianarison, Daria Valente, Paolo Rovara, Giovanna Bonadonna, Olivier Friard, Cristina Giacoma

**Affiliations:** ^1^Department of Life Sciences and Systems Biology, University of TorinoTorino, Italy; ^2^Department of Primatology, Max Planck Institute for Evolutionary AnthropologyLeipzig, Germany; ^3^Département de Paléontologie et d'Anthropologie Biologique, Faculté des Sciences, Université d'AntananarivoAntananarivo, Madagascar

**Keywords:** singing primates, gender differences, lemurs, pitch pattern recognition, musical abilities

## Abstract

A crucial, common feature of speech and music is that they show non-random structures over time. It is an open question which of the other species share rhythmic abilities with humans, but in most cases the lack of knowledge about their behavioral displays prevents further studies. Indris are the only lemurs who sing. They produce loud howling cries that can be heard at several kilometers, in which all members of a group usually sing. We tested whether overlapping and turn-taking during the songs followed a precise pattern by analysing the temporal structure of the individuals' contribution to the song. We found that both dominants (males and females) and non-dominants influenced the onset timing one another. We have found that the dominant male and the dominant female in a group overlapped each other more frequently than they did with the non-dominants. We then focused on the temporal and frequency structure of particular phrases occurring during the song. Our results show that males and females have dimorphic inter-onset intervals during the phrases. Moreover, median frequencies of the unit emitted in the phrases also differ between the sexes, with males showing higher frequencies when compared to females. We have not found an effect of age on the temporal and spectral structure of the phrases. These results indicate that singing in indris has a high behavioral flexibility and varies according to social and individual factors. The flexible spectral structure of the phrases given during the song may underlie perceptual abilities that are relatively unknown in other non-human primates, such as the ability to recognize particular pitch patterns.

## Introduction

It is an open question whether the human ability to produce and perceive sequences of rhythmic sounds arose in an early or later stage in human evolution. Sequences of rhythmic sounds are the core of the musical melodies we listen to in our everyday life, and there is questioning whether we may find primitive forms of music in other species (Brown, 2000; Geissmann, 2000; Merker, 2000). As remarked by Ravignani et al. (2014) temporal properties of animal acoustic behavior should have a primary role in the comparison between human musicality and animal sounds. In animals, there is a wide array of displays that may be well-described with the definition of rhythm by McAuley (2010; see also Toussaint, 2013), “the serial pattern of durations marked by a series of events.” In animal vocal sequences, these “events” are sounds (units) and silences (silent intervals).

Timing and synchronization play a crucial role in human and animal communication (Bowling et al., 2013; Ravignani et al., 2014). From katydids (Greenfield and Roizen, 1993) to fiddler crabs (Blackwell et al., 2006), to amphibians (Klump and Gerhardt, 1992), the temporal organization of acoustic signals has an important part in mediating interactions between individuals and mate choice. Previous studies have shown that generation of rhythmic sound is common for most apes, as what has been termed as drumming (Schaller, 1963) has been found in chimpanzees (*Pan troglodytes*, Goodall, 1986; Nishida, 2011; Babiszewska et al., 2015), bonobos (*Pan paniscus*, de Waal, 1988; Kugler and Savage Rumbaugh, 2002), and gorillas (*Gorilla gorilla*, Schaller, 1963). These sounds can be produced either by pounding with hands and/or feet on external objects or their body and are common in both captive and wild animals (Arcadi et al., 1998, 2004). However, the ability to produce a rhythmic pattern of acoustic signals does not necessarily correspond to the capacity to coordinate sound production (Fitch, 2013). As suggested by Fitch (2006a,b) and Patel (2008), joint coordination in non-human species appears widespread in sound-mimicking birds (*Cacatua galerita*, Patel et al., 2009; *C. galerita* and *Psittacus erithacus*, Schachner et al., 2009; *Melopsittacus undulatus*, Hasegawa et al., 2011) and can extend to sea lions (*Zalophus californianus*, Cook et al., 2013). Studying chorusing dynamics may be of critical importance to understand the flexibility of the individual timing during group displays and the adaptive functions of rhythm (Ravignani et al., 2014). Most studies suggest that monkeys do not perceive a beat and thus they cannot synchronize their movements with it (*Macaca mulatta*, Zarco et al., 2009; Honing et al., 2012), although a certain degree of behavioral coordination between individuals can found in the chorusing of wild chimpanzees (Fedurek et al., 2013a) and the ability of auditory synchronization has been found in captivity (Hattori et al., 2013). Observations of chimpanzees seeking objects with particular resonant properties and then using them repeatedly to drum also suggested a link between the auditory and motor systems in non-human primates (reported by Fitch, 2012).

Apart from temporal patterns, spectral properties also played a major role in the comparison between human musicality and animal vocal behavior. Previous works focused on the fact that non-human species may have a higher capacity for the temporal processing of sounds and lower sensitivity for the spectral harmonicity (*Chinchilla laniger*, Shofner and Chaney, 2013; *Callithrix jacchus*, Pistorio et al., 2006). Studies showed that many non-human primate species, in contrast to humans, did not show considerable differences in average voice pitch between sexes (see Ey et al., 2007 for a review). Patel (2008) suggested that the lack of sex dimorphism in pitch and the limited ability of non-human primates to recognize relative pitch patterns could indicate that sensitivity to pitch changes may be uniquely human, and it may have had had a critical role in the evolution of human musical abilities.

In non-human primates, group calling may have a role in communicating group cohesiveness and in advertising the occupation of a territory (Marler, 2000). Both these functions fit well with the proposed social bonding theory of the evolution of music (Dunbar, 1996) and are crucial for the regulation of territorial ranging patterns and group dynamics (Geissmann, 2002; Gamba, 2014). Non-human primates use song to advertise resource holding potential, to reduce the probability of encounters by regulating group movements in the forest, and to resolve group encounters avoiding physical fights (Mitani, 1985; Cowlishaw, 1996). These findings suggest the existence of neural capacity of advanced sound localization processes in non-human primate species producing songs (Brown, 1982; Maeder et al., 2001).

A quantitative, rigorous investigation of non-human primate singing displays may cast new light about the factors affecting individual singing during chorusing. It also may help in identifying the selective pressures that may have led to the evolution of this trait only in Indriidae, Tarsiidae, Callicebinae, Hylobatidae, (Deputte, 1982; Haimoff, 1983; Geissmann, 2000) and may provide insights into the improvement of these abilities during human evolution.

We investigated the rhythmic abilities of a Strepsirrhini species. Strepsirrhines are primates whose last common ancestor with humans is currently dated back between 64 and 87.2 million years ago^1^. There is a single singing lemur species, *Indri indri* (Gmelin, 1788). The indri lives in the mountain rainforests of Madagascar, where its howling cries can be heard at a distance up to 2 km (Pollock, 1986). The social organization of indri is based on a reproductive pair where the adult female is dominant over the adult male although the level of intra-group competition is low (Pollock, 1975, 1977). Usually a male, whose relatedness with the adult pair is unknown, is present in the social group, and group size usually varies between two and six animals (Torti et al., 2013). The limited number of adult individuals in a group suggested that intrasexual dominance is age-related (Pollock, 1977, 1979).

**Figure 1:**
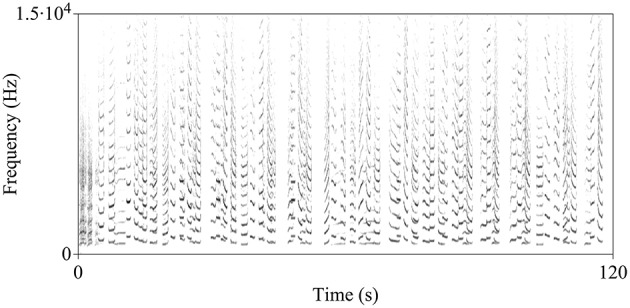
**Spectrogram of the indris' song**. In this particular song recorded in the Maromizaha Forest, a reproductive pair is singing with a male offspring (Group 1 MZ).

The song of the indris is a long sequence of vocal emissions (units) separated by silent gaps and organized in phrases (Figure 1; Thalmann et al., 1993). The indris emit harsh roars at the start of the song, followed by long and scarcely modulated units and, finally, a pattern of descending phrases, which are series of two to six units given with a slightly descending frequency pattern (Thalmann et al., 1993; Sorrentino et al., 2013; Torti et al., 2013). Within the species vocal repertoire, the song is the acoustic display covering the widest range of pitch and all group members aged 2 years and above participate in the song (Maretti et al., 2010). The songs serve to inform neighboring groups about the occupation of a territory and to defend a territory actively during group encounters. They also have a cohesion function (Pollock, 1986; Torti et al., 2013) and are likely to mediate the formation of new groups (Pollock, 1986; Giacoma et al., 2010). It is not clear whether the song may attract partners, but Bonadonna et al. (2014) suggested that, given the scarcity of group encounters, singing may also mediate extra-pair copulation, allow finding a mating partner, and the formation of new groups.

The indri songs are organized behavioral displays where each caller has a precise pattern. Following the frame proposed by Ravignani et al. (2014), we could define the indri songs as the combination of individual aperiodic songs, which shows a complex, uncoupled chorusing of two or more signallers. The calls in the song can be given alternated or simultaneously, with absent, partial, or complete overlap. These characteristics make the indri an excellent model to investigate singing coordination and rhythmic abilities in a non-human species.

Our first aim was to examine coordination during singing between male and female indris. The study of the structure of duetting displays in birds led to two alternative hypotheses. One is that temporal coordination is an honest signal of the coalition quality of the individuals involved (Hall and Magrath, 2007). A coordinated duet is likely to be emitted by an established pair and is more threatening for neighbors than an uncoordinated duet (Brumm and Slater, 2007). A second hypothesis refers to studies demonstrating that temporal coordination may arise when individuals adjust their signals to minimize overlap with conspecifics (Tobias and Seddon, 2009). As the indris form cohesive, territorial pairs, and their songs have a role in advertising territorial occupancy (Torti et al., 2013), we predicted that the reproductive pair would synchronize during singing in most of the songs. Snowdon and Cleveland (1984) showed that pygmy marmosets (*Cebuella pygmaea*) used calls antiphonally to maintain contact, following an individual-specific pattern and a system of rules. Few studies concentrated on primate turn-taking and overlapping during singing. Although a universal pattern cannot be described, studies on members of the family Hylobatidae showed that in sexually dimorphic species, males and females tend to avoid overlapping of their singing, whereas in species where morphological dimorphism is absent singers tend to overlap (Deputte, 1982). From these observations, we predicted that indris, which are not sexually dimorphic and live in socially monogamous groups as gibbons, would overlap during singing. The degree of overlap has been rarely quantified, but the studies of Merker and Cox (1999, *Nomascus gabriellae*) and Koda et al. (2013, 2014; *Hylobates agilis, Hylobates lar*) suggested that juvenile gibbons may overlap more often with adults, especially with adult females. Therefore, our prediction is that gender and dominance would affect the singing displays, in particular, non-adult indris overlapping more with the adults comparing to how much the adults overlap each other.

Our second objective was to identify whether the rhythmic structure of the indris differed between sexes and phrases and to show the developmental dynamics of rhythm in indris. Sasahara et al. (2015) demonstrated that rhythm development in birds shows high rates of change during early stages and then slowly refines toward maturity. Our prediction was that the rhythm of the indris' song phrases differed between age classes.

Our third objective was to investigate pitch variation within and between sexes to understand how sex effects on spectral properties of the indri's vocal signals and complement the results on the temporal patterns. We predicted that indris, which are size monomorphic and monochromatic, would lack marked sexual differences in pitch as it has been shown in most of the non-human primate species (Ey et al., 2007). Thus, we expect indris not to differ markedly in fundamental frequency between sexes and that pitch patterns presented during the song are analogous akin in both genders.

## Materials and methods

### Study subjects and recordings

We studied 21 groups living in four different areas of dense tropical forest in Madagascar: seven groups in the Analamazaotra Reserve (Andasibe-Mantadia National Park, 18° 56′ S, 48° 25′ E), two groups in Mantadia (Andasibe-Mantadia National Park), three groups in the Mitsinjo Station Forestière (18° 56′ S, 48° 24′ E), and nine groups in the Maromizaha Forest (18° 56′ 49″ S, 48° 27′ 53″ E). We collected data in the field every year between September and December, from 2004 to 2014, for a total of 30 months. We observed one group per day from 06:00 a.m. to 1:00 p.m. Natural marks allowed identifying each indri individually. The reproductive life of indris begins at 6–7 years of age (Pollock, 1977), thus, we labeled all the indris aged six or more as “adults,” and all the animals aged between two and five as “non-adults.” The reproductive individuals as reported by the guides and the genetic analyses (Bonadonna, unpublished data) were indicated as “dominant,” all other members were labeled “non-dominant.”

Recordings were made using Sennheiser ME 66 and ME 67 and AKG CK 98 microphones. The microphone output signal was recorded at a sampling rate of 44.1 kHz using a solid-state digital audio recorder (Marantz PMD671, SoundDevices 702, Olympus S100, or Tascam DR-100MKII 24 bit/96 kHz). All utterances were recorded at a distance from 2 to 10 m since all the study groups were habituated, and all efforts were made to ensure that the microphone was oriented toward the vocalizing animal. All recordings were made without the use of playback stimuli, and nothing was done to modify the behavior of the indris. We recorded “advertisement” songs (Torti et al., 2013), consisting of duets and choruses, with a maximum of six individuals singing the same song. When in the field, we had one observer per individual indri in a group. We used Focal animal sampling (Altmann, 1974) that allowed the attribution of each vocalization to a signaller.

We recorded a total of 496 songs. To investigate the coordination during singing, we measured the amount of overlap between two singers of the same group (hereafter, co-singing) and the timing in which each unit started being emitted during a song. For the co-singing analysis, we used 223 songs of 45 individuals (15 dominant adult males, 15 dominant adult females, 15 non-adult indris (11 males, four females). The timing was analyzed in 119 songs and 40 individuals (18 dominant adult males, 14 dominant adult females, three non-adult males, one non-adult female). For the analysis of the rhythmic pattern of the descending phrases (hereafter, DPs), we considered phrases consisting of two (hereafter, DP2), three (DP3), and four (DP4) units extracted from 475 songs and 57 individuals: 23 dominant adult males, 20 dominant adult females, seven non-adult males, three non-adult females. We investigated pitch variation of 1919 DP2s, 2182 DP3s, and 1046 DP4s extracted from 1060 individual song contributions. The sampling included phrases emitted by 25 dominant adult males, 21 dominant adult females, 17 non-dominant non-adult indris (10 males and 7 females).

### Acoustic analyses

**Figure 2:**
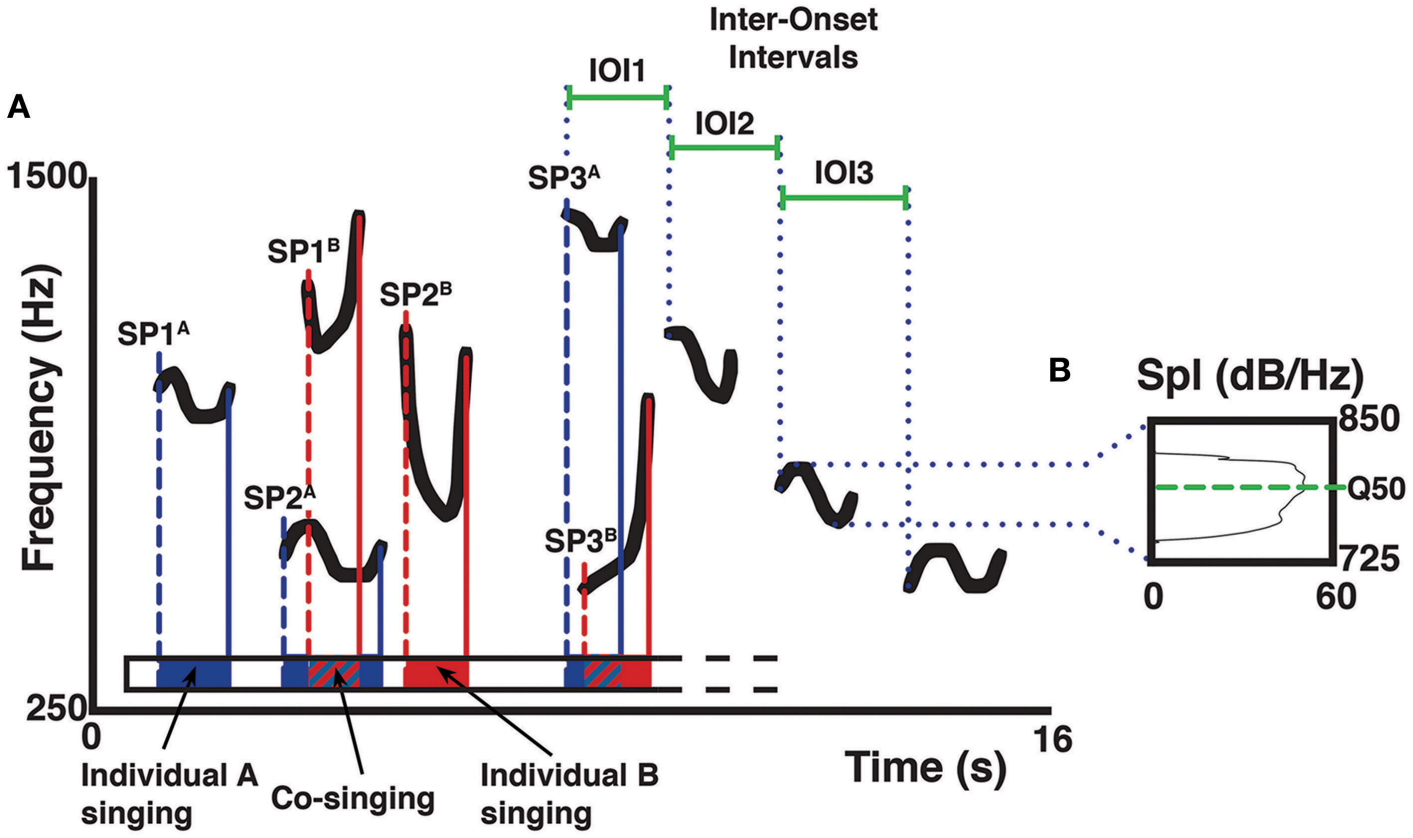
**Schematic representation of a spectrogram (A) describing acoustic parameter collection on the isolated pitch of a song**. Letters A and B mark different singers, letters SP mark the starting points of a unit (1, 2, 3…) in the song. The color bars indicate the starting and final points of the units given by two different indris (e.g., blue for a male; red for a female). Duration of the units is reflected in the schematized Praat textgrid as an interval of the same color, where solid colors indicate non-overlapping parts and striped patterns indicate co-sung portions. Duration of the IOIs of a descending phrase is marked by solid green bars. In the spectrum **(B)** of the third unit (in a descending phrase of four units), the green dotted line marks the frequency corresponding to the upper limit of the second quartile of energy in the spectrum (Q50). The sound spectrum displays sound pressure level (Spl) on the x-axis, frequency on the vertical axis.

We edited segments containing indri's songs using Praat 5.3.46 (Boersma and Weenink, 2008), and we saved each song in a single audio file (in WAV format). Using our field notes and video recordings, we identified and selected the individual contribution of each singer, and we saved this information in a Praat textgrid. We then merged textgrids of all the singers of a song to quantify the co-singing between individuals, and the portions of non-overlapping singing (those in which only one singer was vocalizing). In the case of co-singing of three indris we added that percentage to each dyad involved. We expressed the overall co-singing and non-overlapping as a percentage of the total song duration (Figure 2). The duration of co-singing and non-overlapping segments of each song, as well as the timing of the starting points of each song unit, were saved in Praat and exported to a Microsoft^©^ Excel spreadsheet (Gamba and Giacoma, 2007; Gamba et al., 2012). We used the duration of overlapping contributions of each particular pair of individuals to quantify the amount of co-singing between adults and non-adults of both sexes and to calculate the ratio of co-singing within the contribution of an individual to the song. We then used the timing of the starting points of each song unit to understand whether the timing of a singer influenced another indri's song timing. Following Sasahara et al. (2015), we quantified the inter-onset intervals (IOI) of two adjacent units and used it as a proxy for the rhythmic structure of a phrase.

We processed the DPs to extract the pitch of the focal animal in Praat, discarding the contribution of other singers and the background noise. We analyzed pitch variability by setting a frequency range from the minimum to the maximum of each unit in a DP and then calculating the frequency value at the upper limit of the second (Q50) quartile of energy (Figure 2).

### Statistical analyses

We ran the General Linear Mixed Models (GLMMs) using the lme4 package (Bates et al., 2015) in R (R Core Team, 2015; version 3.2.0).

The model we used to investigate IOI variation included the duration of IOI as the response variable, IOI type (IOI1, IOI2, or IOI3), sex, age cohort (adult vs. non-adult), and DP type (DP2, DP3, and DP4) as fixed factors and group ID, song ID, site ID, and individual ID as random factors.

To analyse the co-singing, we used a model where the duration of the overlap between two singers was the response variable. The predictors were the duration of the individual contribution, song duration, the number of singers, sex of the focal animal, sex of the co-singer, the status of both the focal animal and the co-singer (identified as dominant or non-dominant in their natal groups). We used group ID, song ID, individual ID (for both the focal and the co-singer), and site ID as random factors. Since, we predicted that the degree of overlap during the song of one individual would be influenced by the sex and the status of its co-singer, we included in this model two interactions: one between the sex of the focal individual and the sex of the co-singer, and another between the status of the focal and the status of the co-singer.

For both models, we verified the assumptions that the residuals were normally distributed and homogeneous by looking at a qqplot and the distribution of the residuals plotted against the fitted values (a function provided by R. Mundry). We excluded the occurrence of collinearity among predictors by examining the variance inflation factors (*vif* package; Fox and Weisberg, 2011). To test the significance of the full model (Forstmeier and Schielzeth, 2011) we compared it against a null model comprising the random factors exclusively, by using a likelihood ratio test (Anova with argument test “Chisq”; Dobson, 2002). Then, we calculated the P values for the individual predictors based on likelihood ratio tests between the full and the respective null model by using the R-function “drop1” (Barr et al., 2013). We used a multiple contrast package (*multcomp* in R) to perform all pairwise comparisons for the levels of each factor with the Tukey test (Bretz et al., 2010). We adjusted all the *p*-values (*p*_adj_) using the Bonferroni correction. We reported estimate, standard error (*S.E.*), *z*- and *p*-values for the Tukey tests.

**Figure 3:**
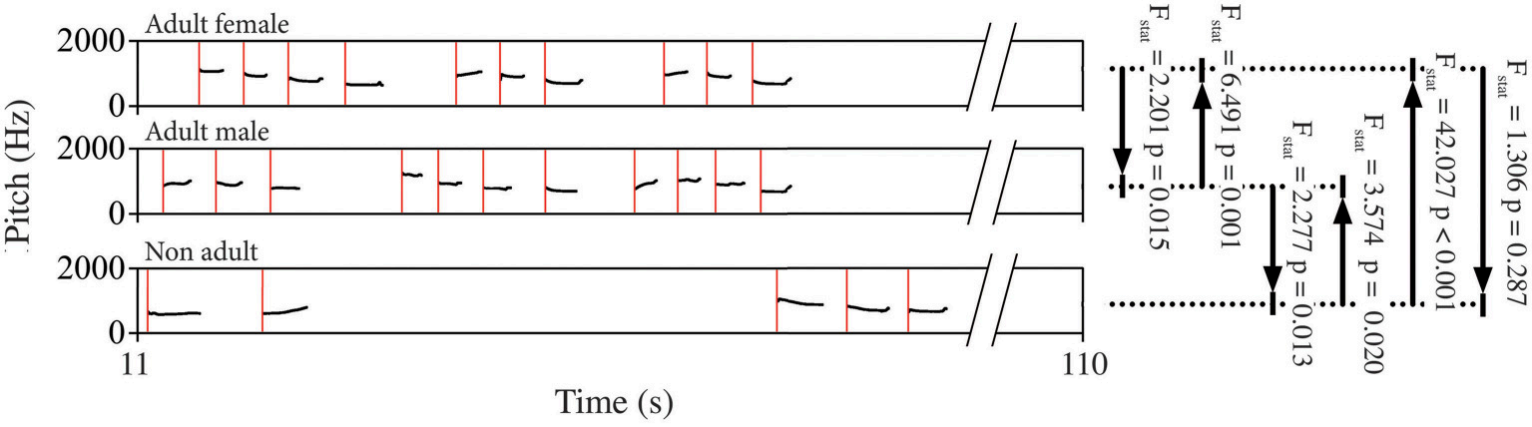
**Schematic representation of a spectrogram showing the pitch contour of a portion of the song of three different indris**. Red lines mark the starting point of each unit, which were entered in the Granger causality test. Black lines and arrows indicate the dyad and the direction of the test, for which we reported *F* statistics (*F*_stat_) and *p*-values (*p*) as examples.

The predictive power of the song unit timing in one individual over another was evaluated using the Granger Casuality test (Granger, 1969). We computed the bivariate Granger causality test in two directions for each dyad of indris singing in a chorus (Brandt et al., 2008; Wessa, 2013) tracking whether they were males, females or non-adults. We used a lag-4 analysis (*MSBVAR* package v.0.9-1 in R) and considered significant those analyses showing *p*-values below 0.05 (Figure 3). We then calculated the percentage of significant *p*-values on the total of the songs, overall and for each particular dyad. We then average the results per type of dyad. We did not tested dyads of two subadults because of the small sample size.

To analyse the sex dimorphism in pitch, we used four GLMMs where the frequency at the upper limit of the second quartile of energy in the spectrum Q50 was the response variable. We run a model for each unit in a DP. The predictors were sex, status (dominant or non-dominant), age cohort (adult vs. non-adult), and DP type (DP2, DP3, and DP4) as fixed factors and group ID, song ID, site ID, and individual ID as random factors. We verified the assumptions and the significance of the models as explained for the models above.

We presented the average variation of IOIs and the average variation of pitch between different units by calculating average individual means, first at the song level, then at the individual level, and finally by sex.

## Results

### Overlapping between singers

We found a considerable amount of co-singing in all the songs (average individual mean 28.10% ± 7.64, *N* individuals = 45). The average total duration of the song was 113.188 ± 39.682 s while the duration of an individual's phonation during the song was 30.132 ± 10.301 s (29.73% ± 11.24). The average total co-singing during the song was 8.019 ± 3.587 s. The full model significantly differed from the null model (χ^2^ = 144.080, *df* = 9, *P* < 0.001). Since the interaction between the sexes of the singing pair was not significant, we ran a reduced model excluding such interaction. The results of such reduced model are in Table 1. The duration of co-singing increased significantly with the duration of the individual contribution, but not with song duration itself. The number of singers in a song significantly decreased the amount of co-singing between two singers in a song. Moreover, co-singers' status significantly affected the response variable, with increased co-singing when two non-dominant individuals sang together. We have also found that the two dominant individuals in a group co-sing significantly longer than a dominant and a non-dominant indri (Tuckey test, estimate = −1.1546; *S.E.* = 0.2070; *z* = −5.578; *p*_adj_ < 0.001) and than non-dominants singing together (Tuckey test, estimate = −1.3719; *S.E.* = 0.3598; *z* = −3.813; *p*_adj_ < 0.001). The model did not detect any effect for the sex of the co-singers.

**Table 1:** **Influences of the fixed factors on cosinging duration (s); results of the reduced model, including only the significant interaction (full vs. null: chisq = 144.080, *df* = 9, *P* < 0.001)**.

	**Estimate**	***SE***	***df***	**χ^2^**	***P***
(Intercept)	2.100	0.312	^a^	^a^	^a^
Duration of the individual contribution	0.018	0.002	1	73.103	< 0.001
Song duration	0.001	0.001	1	1.398	0.237
Number of singers	−0.216	0.071	1	8.992	0.003
Focal sex (Male)^b^^,^^c^	−0.029	0.154	1	0.033	0.856
Cosinger sex (Male)^b^^,^^c^	−0.093	0.154	1	0.335	0.563
Focal class (Non-dominant)^b^^,^^c^	−0.840	0.187	^d^	^d^	^d^
Cosinger class (Non-dominant)^b^^,^^c^	−1.138	0.184	^d^	^d^	^d^
Focal class (Non-dominant): Cosinger class (Non-dominant)^b^^,^^c^	0.922	0.362	1	5.955	0.015

a Not shown as not having a meaningful interpretation.

b Estimate ± SE refer to the difference of the response between the reported level of this categorical predictor and the reference category of the same predictor.

c These predictors were dummy coded, with the “Focal sex (Female),” “Cosinger sex (Female),” “Focal class (Dominant),”and “Cosinger class (Dominant)” being the reference categories.

d Not shown, as the interaction between these predictors is significant.

### Gender and status influence on the singing pattern

**Figure 4:**
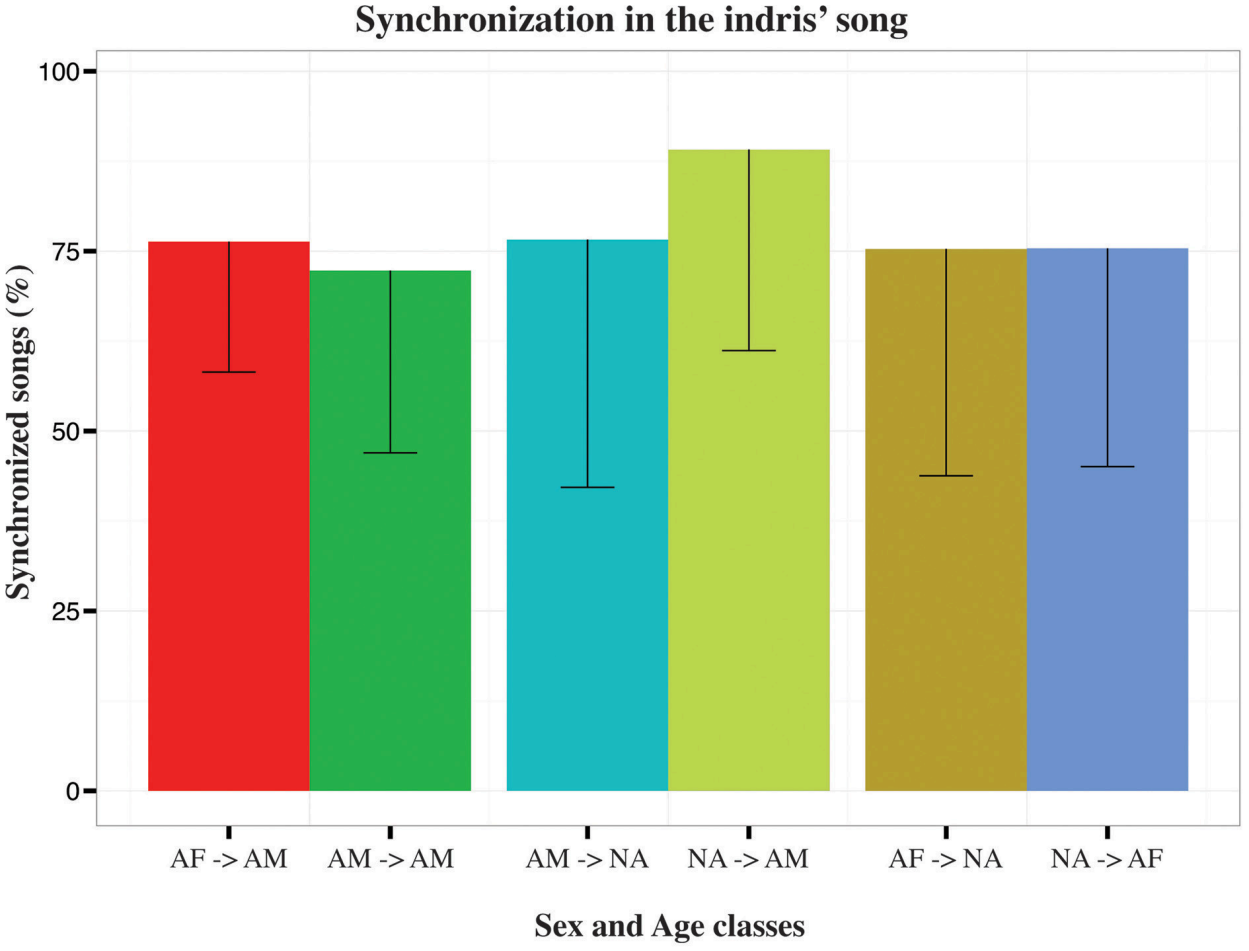
**Bar plot of the average percentage of synchronized songs in the indris**. Capped lines represent negative Standard Deviation. Each bar indicates the direction of the Granger causality test for each type of dyad (AF, adult females; AM, adult males, NA, non-adults).

We asked whether singing of a particular indri influenced the contribution of another animal to the song. Applying the causality test between the timing of the onset in the individual contributions, we found an effect of the adult male singing on the pattern of the adult female in 68% (*N* = 94) of the songs (902.01 < *F*_stat_ < 1071.97; 0.001 < *p*_adj_ < 0.039). The timing of the adult female was useful to forecast when the adult male was singing in 73% (*N* = 91) of the songs (9.53 < *F*_stat_ < 10.44; 0.001 < *p*_adj_ < 0.043). The non-adults in a group influenced adult male and adult female singing in 94% (*N* = 47; 78.20 < *F*_stat_ < 10.08; 0.001 < *p*_adj_ < 0.036) and 75% (*N* = 63; 9315.05 < *F*_stat_ < 105.22; 0.001 < *p*_adj_ < 0.042) of the songs respectively. We found an effect on non-adults in 81% (*N* = 57) of the songs for the contribution of the adult female (90.86 < *F*_stat_ < 10.00; 0.001 < *p*_adj_ < 0.046) and 78% (*N* = 46) of the songs for the adult male (9.97 < *F*_stat_ < 10.54; 0.001 < *p*_adj_ < 0.030). We also analyzed data by considering each pair and dyad. We found that non-adults effect on the adult males was 89.10% ± 27.93 (N = 13) and all other combinations ranged between 72.31% ± 25.34 and 76.60% ± 34.42 (Figure 4).

### Rhythmic differences between sexes and age classes

**Figure 5:**
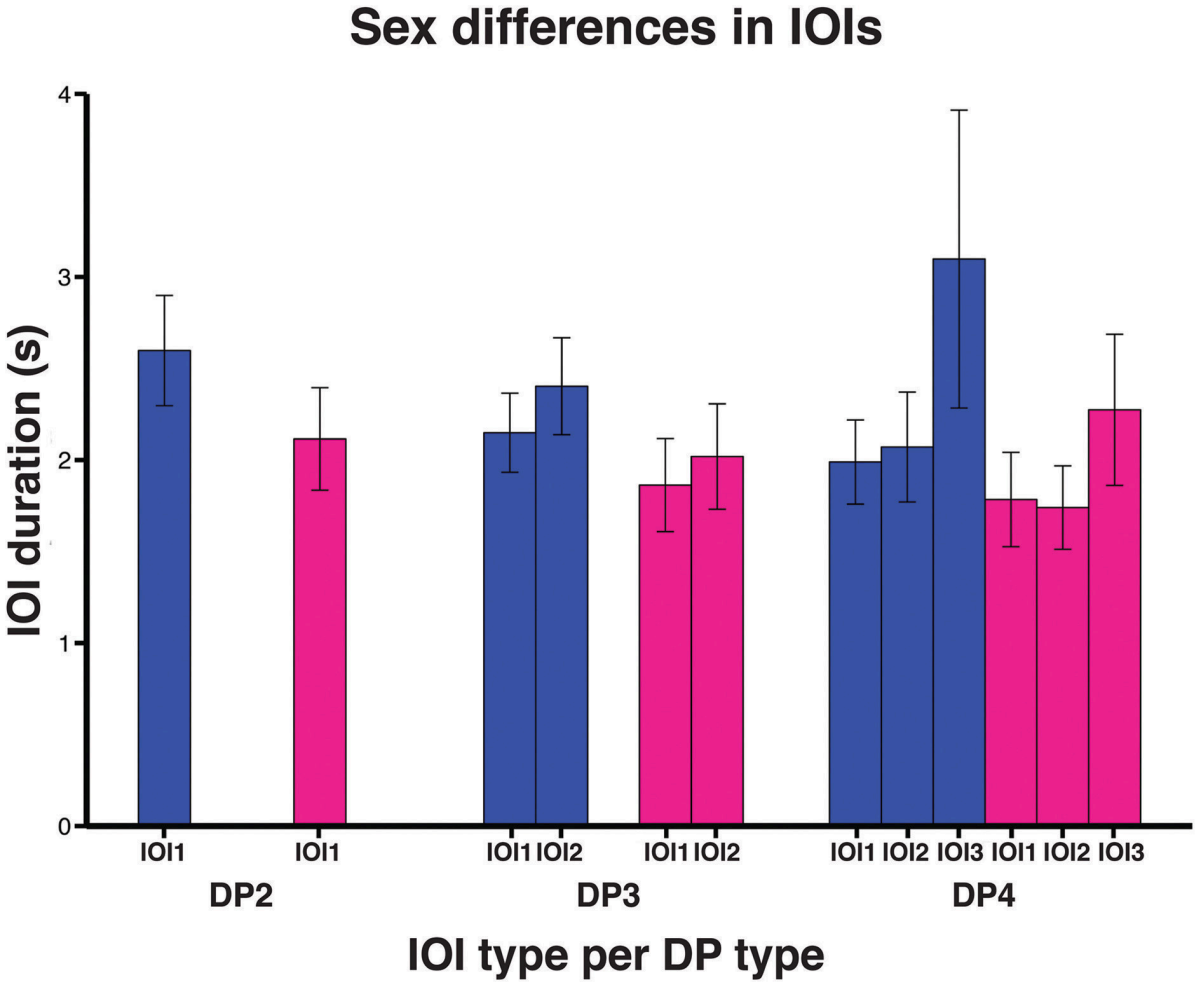
**Bar plot of the average IOI duration between DP types in the sexes (males in blue, females in magenta)**. Capped lines represent ± Standard Deviation.

We then investigated to what extent sex and age affected indris' singing rhythm. The full model significantly differed from the null model (χ^2^ = 144.080, *df* = 9, *P* < 0.001). We found that the IOI type significantly affected its duration, in particular both the types IOI2 and IOI3 were significantly longer than IOI1 (Table 2). IOI2 was also significantly shorter than IOI3 (Tuckey test, estimate = 0.063; *S.E.* = 0.014; z = 44.58; *p* < 0.001). The IOI duration significantly decreased at the increase of the number of units in the DP (Figure 5; Table 2). In particular, IOIs of DP2s are longer than those in the other DP types, but we found that also IOIs in the DP3s are significantly longer than those in DP4s (Tuckey test, estimate = −0.179; *S.E.* = 0.010; *z* = −17.74; *p*_adj_ < 0.001). We have also found a significant effect of sex, where males showed longer IOIs (Table 2) when compared to females. We found no effect of age cohort (Table 2).

**Table 2:** **Influences of the fixed factors on IOI duration (s); results of the full model (full vs. null: chisq = 2966.748, *df* = 6, *P* < 0.001)**.

	**Estimate**	***SE***	***df***	**χ^2^**	***P***
(Intercept)	2.094	0.049	^a^	^a^	^a^
IOI Type (2)^b^^,^^c^	0.157	0.009	2	2640.061	< 0.001
IOI Type (3)^b^^,^^c^	0.785	0.014	2		
Sex (Male)^b^^,^^c^	0.405	0.060	1	32.848	< 0.001
Age Cohort (Not adult)^b^^,^^c^	0.043	0.029	1	2.152	0.142
DP TYPE (DP3)^b^^,^^c^	−0.279	0.011	2	1228.102	< 0.001
DP TYPE (DP4)^b^^,^^c^	−0.458	0.013			

a Not shown as not having a meaningful interpretation.

b Estimate ± SE refer to the difference of the response between the reported level of this categorical predictor and the reference category of the same predictor.

c These predictors were dummy coded, with the “IOI Type (1),” “Sex (Female),” “Age Cohort (Adult),” and “DP TYPE (DP2)” being the reference categories.

### Pitch variation patterns

**Figure 6:**
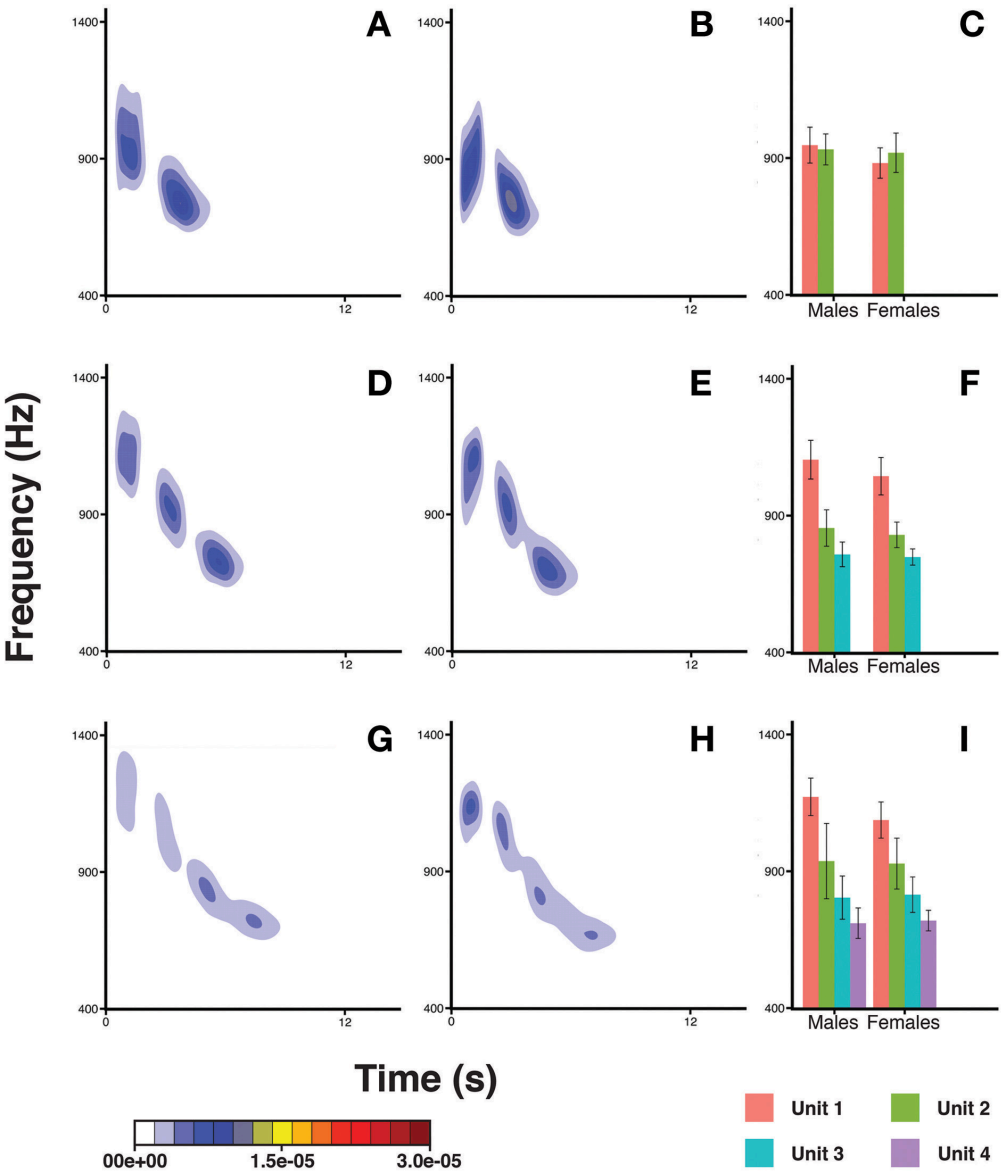
**Density plots obtained in R (*MASS* package) for male descending phrases DP2s (A), female DP2s (B), male DP3s (D), female DP3s (E), male DP4s (G), and female DP4s (H)**. The bar plots show the average frequency corresponding to the upper limit of the second quartile of energy in the spectrum (Q50) for males and females, for DP2s **(C)** DP3s **(F)**, and DP4s **(I)**. Units within a DP are indicated by different colors. Capped lines represent ± Standard Deviation.

The pitch pattern of the units in a DP showed remarkable inter- and intra-individual frequency variation (Figure 6). We found that the frequency value corresponding to the second quartile of energy Q50 was significantly higher in males (Table 3) than in females. The Q50 of unit 1 was significantly higher than those of Unit 2, 3, and 4 (Table 3), which appeared descending in the frequency value Q50 along the DP (−259.485 < Estimate < −107.059; 3.819 < *S.E.* < 9.899; −39.92 < *z* < −10.81; all Ps < 0.001). The Q50 also differed significantly between DP types. DP4 and DP3 showed higher values than DP2 (Table 3), and also DP4 showed greater values than DP3 (Tuckey test, estimate = 68.322; *S.E.* = 5.264; *z* = 12.98; *p* < 0.001).

**Table 3:** **Influences of the fixed factors on Q50 frequency (Hz); results of the full model (full vs. null: chisq = 4330.685, *df* = 7, *P* < 0.001)**.

	**Estimate**	***SE***	***df***	**χ^2^**	***P***
(Intercept)	967.260	13.343	^a^	^a^	^a^
Unit (2)^b^^,^^c^	−104.697	2.748	3	4193.205	< 0.001
Unit (3)^b^^,^^c^	−257.122	3.819			
Unit (4)^b^^,^^c^	−364.181	9.780			
Sex (Male)^b^^,^^c^	36.067	6.479	1	21.804	< 0.001
Age Cohort (Non-adult)^b^^,^^c^	9.396	6.652	1	1.984	0.159
DP TYPE (DP3)^b^^,^^c^	33.106	2.857	2	375.086	< 0.001
DP TYPE (DP4)^b^^,^^c^	101.427	5.430			

a Not shown as not having a meaningful interpretation.

b Estimate ± SE refer to the difference of the response between the reported level of this categorical predictor and the reference category of the same predictor.

c These predictors were dummy coded, with the “Unit (1),” “Sex (Female),” “Age Cohort (Adult),” and “DP TYPE (DP2)” being the reference categories.

## Discussion

### Coordination and overlapping during singing

Despite a majority of non-overlapping singing, an important part of the individual song was co-sung with another member of the social group with a positive effect of the duration of the singer's contribution rather than overall song duration. We found support for our prediction that indris, being not sexually dimorphic, would overlap during singing, in agreement with what postulated by Deputte (1982) on the Hylobatidae. This finding appears to confirm what previous studies have shown for gibbons. The sex-specific individual song contributions may indeed serve different functions and therefore, may be under different selective pressures (Cowlishaw, 1992; Geissmann, 2002). At the same time, the overlap has an adaptive value because it may have a role in signaling group cohesion and resource holding potential to conspecifics (Torti et al., 2013).

Describing the temporal properties of the indris' singing, we found that the singer's and co-singer's sex did not affect co-singing duration, showing that not only indris of the two sexes participate equally to the song (Giacoma et al., 2010) but they also similarly co-sung with conspecifics of the opposite sex. We found instead that being dominant or non-dominant affected co-singing rates during a group song, in agreement with what Cowlishaw (1992) suggested about the duration of solo bouts in gibbons (see also Mitani, 1984, 1987; Dallmann and Geissmann, 2009). In the indris, solo songs are exceedingly rare. Giacoma and colleagues (unpublished data) recorded three songs emitted by a single young adult indri male during a sampling time in which over 600 duets and group choruses were recorded. Thus, we can suppose that the indris chorusing may itself play a role in the competition among paired and unmated males, and that conspecifics may assess males' (and females') characteristics from their collective singing (Torti et al., 2013).

The fact that indris showed overlapping avoidance in between dominants and non-dominants (which are often sub-adults in our sampling) and more frequent overlapping between adult males and females marks a difference to what is known for gibbon songs. Adult male gibbons and females tended to alternate their calls and immature individuals frequently overlap (Merker and Cox, 1999; Koda et al., 2013), but a different scenario emerged from our findings. The fact that adults singing together showed a significantly longer overlap falsified our second hypothesis that co-singing rates in these species are higher between non-dominants and dominants. Our results suggest that overlap between adults can indeed serve inter-group communication as suggested by previous studies (Merker, 2000). Co-singing may correspond to louder signals, and overlapping of the paired mates may serve to maintain a territory. Non-overlapping singing may provide the advantage of advertising the resource holding potential of the group, but overlapping another conspecific may represent a cost for an individual singer, which cannot broadcast its individuality. It makes sense that non-dominant individuals tend to co-sing less than paired, dominant indris. Non-dominant indris may attempt to maximize their solitary singing during the chorus, to advertise their fighting ability to conspecifics of other groups and their individuality to potential mates (Cowlishaw, 1992).

Studying chorusing dynamics, we found that differences in co-singing reflect differences in coordinating the emissions of units during the song. We demonstrated the existence of a coordination of the calls in both dominant and non-dominant individuals, with a consistent influence between the singing of different indris during the song. We found that the coordination between singers was mutual between sexes and age cohorts, but the non-dominants appeared to have an especially strong effect on dominant adult males. Indris within a group coordinated on average more than 70% of their songs to form duets, suggesting that duetting is indeed associated with pair cohesion and the strength of the pair bonds (Geissmann and Orgeldinger, 2000). In indris, as it happens for bird species, duetting may have a crucial role in territory defense but may also have evolved for multiple functions (Dahlin and Benedict, 2013), including the localization of conspecific (Torti et al., 2013; Bonadonna et al., 2014) and providing information about the quality of their pair bond (Merker, 2000; Hall and Peters, 2008; Hall, 2009; Dowling and Webster, 2015).

Unlike what Geissmann (2000) hypothesized for gibbons (2000), the indris' song may also facilitate finding a mate either for an extra-pair copulation (Bonadonna et al., 2014) or to form a new pair (Torti et al., 2013). Thus, the interplay between singers can be particularly meaningful for the non-adults which may attempt to broadcast their individuality and may affect the dominant male singing pattern. We cannot exclude that dominant male singing may contribute to the development of singing non-dominant indris, as it has been found in gibbons (Koda et al., 2013).

Acoustic analyses of indris' vocal behavior during the song may also indicate the ability of precise timing in a particular social display, like the song. A parallel with humans may be found in the study of Bowling et al. (2013) showing that speech timing is more precise when speakers are together with a partner than when the same speaker is alone. Further studies are needed, but the investigation of songs given in different behavioral context showed that animals tended to turn taking more precisely when in visual contact than when they were not (Torti et al., 2013). Moreover, dominant adults may indeed have a synchronization capacity that is developing in younger non-dominants.

### Rhythmic differences in the indris

We identified a system of distinct units produced in sequences in agreement with previous studies (Thalmann et al., 1993; Giacoma et al., 2010; Baker-Médard et al., 2013; Torti et al., 2013; Gamba et al., 2014). We analyzed short phrases consisting of two, three or four units and we found that the rhythmic structure differed within and between descending phrases. Namely, the interval between onsets decreased significantly during a DP, but also differed between DP types.

These differences in the rhythmic structure of descending phrases suggest that indris may be capable of regulating timing, unit duration, and interval duration. This ability appears similar to those shown by the chimpanzees producing a “pant hoot chorus.” In agreement with the findings of Fedurek and colleagues on the chimpanzees, the indris appear to adjust the timing of their emissions (Fedurek et al., 2013a) during the song and to do that to interact vocally with another member of their social group (Mitani and Gros-Louis, 1998). The ability to adjust the emissions within a song has emerged when investigating contextual variation in the acoustic structure of the song (Torti et al., 2013). It may indeed play a role in social interactions within- and between-groups as it has been suggested for chimpanzees' joint hooting (Fedurek et al., 2013b) or agile gibbons' singing (Koda et al., 2013).

We also demonstrated that there is a remarkable difference between males and females, with females showing shorter IOI in all DP types. This sex dimorphism in rhythm is surprising when seen in the light of the indris' social monogamy and external morphology, which would both predict a little dimorphism in the size of the vocal apparatus (Dixson, 2013). Current data on indris' vocal tract morphology is poor, but we found reference to the fact that both males and females possess a dorsal air sac (Grandidier, 1875; Petter et al., 1977). The presence of larger vocal sacs in the male indris could explain the longer IOI observed in all descending phrases. The study on apes showed that there is usually a pronounced sex dimorphism in the size of the vocal sac in the polygynous species (*G. gorilla, Pongo pygmaeus*), which also produce sex-specific calls (Harcourt et al., 1993; Delgado and Van Schaik, 2000). This dimorphism is apparently less marked in the chimpanzees (*P. troglodytes schweinfurthii*) group cohesion pant hoots, which are given by both sexes (Mitani and Nishida, 1993). Recent studies on the howler monkeys (*Alouatta* sp.) confirmed a role of vocal competition and suggested that vocal tract traits have been sexually selected in those forest-living, arboreal species (Dunn et al., 2015). Vocal competition can also occur for indris, where sexual monogamy may occur together with the presence of multiple males and females within a group, can involve extra-pair copulation (Torti et al., 2013; Bonadonna et al., 2014) and where inter-sexual selection may have played a role (Singleton et al., 2009).

We found support for our prediction that IOIs differed between males and females. The results instead falsify the hypothesis that rhythm changes during the indris' development because we failed to find clear changes in rhythm between indris of different age cohorts. These results are in disagreement with previous finding on birds (Saar and Mitra, 2008; Sasahara et al., 2015), although the analysis of the entire song instead of single phrases could lead to different results. However, we are convinced that our findings clearly show that non-adults rhythms did not substantially differ from the adult rhythms. These findings also provide insight into the development of the indris' song showing that when the animals start singing the cognitive processes and the vocal apparatus that produces song are fully developed. Thus, the dynamics of the song, at least at a phrase level, has then a limited plasticity.

### Pitch variation

Our results showed that units emitted sequentially in the DPs differ consistently in frequency, in agreement with the qualitative observations of Thalmann et al. (1993) and Giacoma et al. (2010). The units given during the DPs have a descending frequency on average with remarkable individual variation. We demonstrated that pitch differs between sexes, despite a similar trend in frequency change.

We expected variation within individuals apparently to override sex differences, but the results falsified the prediction that indris lacked marked sexual differences in the pitch of song units. Our findings are in contrast with the general frame reported by Ey et al. (2007) and show that indris present sexual vocal dimorphism. The presence of differences in frequency variation is shown in our study across comparable series of units, and not limited to different unit types, as previously found by Sorrentino et al. (2013).

Indris are sexually monomorphic (Pollock, 1977), and group encounters are rare (Torti et al., 2013). Thus, sex recognition relying on vocal signals is potentially useful and may be indeed encoded both in the rhythmic structure and the frequency of the DP units. The use of song phrases to broadcast sex may be essential during pair formation (Torti et al., 2013) at distances where other communicative signals may be ineffective (Fletcher, 2009).

We support the conclusions of Torti and colleagues suggesting that the song, or part of the song, may be important in sex recognition and for finding mates, but the question of whether indris recognize the sex of an individual listening to its song is still unanswered. As suggested by previous theoretical works, singing in indris is probably the results of several selective pressures that acted differently on the two sexes. Whether indris have a voluntary control over their timing is still unclear and can be further investigated. However, as Gamba (unpublished data) observed in captive siamangs (*Symphalangus syndactylus*, the emission of harsh sounds (“barks” in siamangs, “roars” in indris) may serve as to synchronize the successive emissions of group members (Giacoma et al., 2010; Torti et al., 2013). Then, the song reaches its most consistent portion of the emission of the descending phrases (Torti et al., 2013), which indeed represent an interesting case of timing and pitch variation, a crucial feature of birdsong and human speech (Levinson and Holler, 2014).

The musical ability of animals has been connected to species-specific perceptual templates, which may in some species change according to brain plasticity. However, the extensive evidence of the processes involved in learning concerns bird and humans (Maguire et al., 2000; Kilgard et al., 2001; Anderson et al., 2002) and there is no equivalent evidence for primates. Our knowledge of primates, and especially of “singing primates” is limited to behavioral observations and few experiments. Studies on humans and other mammals demonstrated that learning corresponds to plastic changes in the auditory cortex (Metherlate and Weinberger, 1990; Norton et al., 2005), but it is still unclear whether this can also be the case of non-human primates and can indeed involve processes involved in vocal production learning.

The indris are good candidates for further investigations of the evolution of typical speech features because the turn-taking between individuals, the constant exchange of short vocal units, and the variable degree of overlap are shared trait of modern human communication.

## Author contributions

MG, VT, GB, and CG designed research; MG, VT, VE, RR, DV, GB, and CG performed research; MG, VT, DV, OF, PR, and VE analyzed data; MG, VT, VE, and CG wrote the paper.

### Conflict of interest statement

The authors declare that the research was conducted in the absence of any commercial or financial relationships that could be construed as a potential conflict of interest.

## Footnotes

^1^Strepsirrhines are primates whose last common ancestor with humans is currently dated back between 64 and 87.2 MYA. Estimates may differ according to the methodology used for the phylogenetic reconstruction: 67.1–97.7 MY in Steiper and Young (2006); 64 MY in Chatterjee et al (2009); 87.2 in Perelman et al. (2011).
